# Reckless formalin injection in the eyelid instead of local anesthetic: case report

**DOI:** 10.11604/pamj.2016.24.304.10260

**Published:** 2016-08-10

**Authors:** Tasnim Masmoudi, Mohamed Mahjoub, Youssef Chkirbene, Maher Jedidi

**Affiliations:** 1 Department of Legal Medicine, University Hospital Center Farhat Hached, Sousse, Tunisia; 2 Department of Hospital Hygiene, University Hospital Center Farhat Hached, Sousse, Tunisia

**Keywords:** Reckless injection, formalin, anesthetic, damage, reparation, legal

## Abstract

Accidental injection of formalin is certainly rare, but it has serious consequences in terms of morbidity and mortality. We report a case of severe ophthalmic damage due to an accidental formalin's injection in the eyelid of a three-year-old child presenting with congenital ptosis's surgical repair of her left eye. This accident has damaged the orbital region and led to upper left eyelid's necrosis, eyeball's deformation and ipsilateral cataract. In terms of our observation, we discussed consequences of this rare type of accident, and its systematic and loco-regional effects. We tried also to explain these manifestations by analyzing the metabolism process of formalin in the human body. Finally, a medico-legal implication of such accident has been illustrated according to the Tunisian law (Penal Code of the Tunisian republic 'PCT').

## Introduction

Formalin is a worldwide used aqueous solution. It is composed of 35 to 40% of stabilized formaldehyde with methanol (8 to 12%) with or without formic acid (0.03%). This caustic and irritating substance is used for its bactericidal, virucidal and conservation capacity (mainly in pathology). Also, it is useful in hygiene and especially disinfection, in therapeutic stomatology (ductal pasta, resins) and industry (manufacturing of plastics, chemical, cosmetics, pharmaceuticals) [1, 2]. There has been a great interest in the toxicity of formaldehyde, and most of the recent concern has centered on formaldehyde's release from urea-formaldehyde foam into the environment to be used in insulation products [1, 3]. Human formaldehyde intoxication has not been adequately characterized, and there are few resources that talk about it. However, it has many side effects. In addition to loco-regional consequences due to direct exposure by inhalation, skin contact or ingestion, there are systemic effects which can lead to multi-visceral failure [3]. We may notice different manifestations such as gastrointestinal trouble, nasal lesions, laryngeal and broncho-pulmonary trouble, cell lysis and tissue necrosis following injections and atopic signs ranging from hives to anaphylactic shock [4–6]. Thus, formalin morbidity and mortality are recalled to justify the rules, measures and precautions of its use in caring environments [7]. This rare medico-legal case illustrates the consequences of medical malpractice which consists of an accidental injection of formalin in the upper eyelid during surgical repair of a ptosis.

## Patient and observation

A three-year-old oriental female infant with no significant personal and family medical history except a congenital ptosis in the left eye was admitted and was proposed to a surgical repair of her congenital ptosis under local anesthesia. Immediately after the injection of the supposed local anesthetic, the patient complained of progressively increasing severe burning sensation over the lid and also around the left eye. Doctors noted unusual modification of the cutaneous tissue which became less well- vascularized with a hardcover consistence and the levator muscle of the left upper eyelid which became less flexible with chemosis and corneal opalescence. An intentional fault was suspected, and immediately a toxicological exam was required.

**Figure 1:**
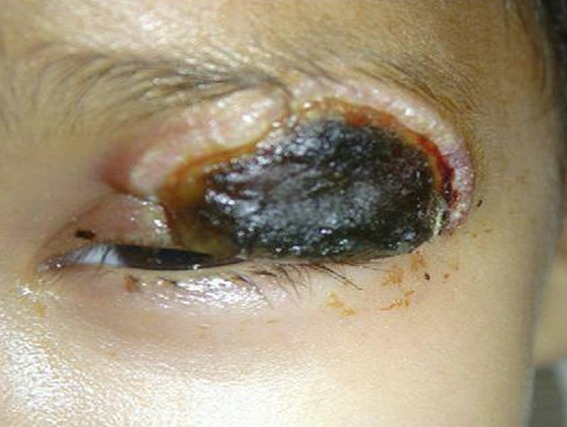
Necrosis of top-left eyelid

**Figure 2:**
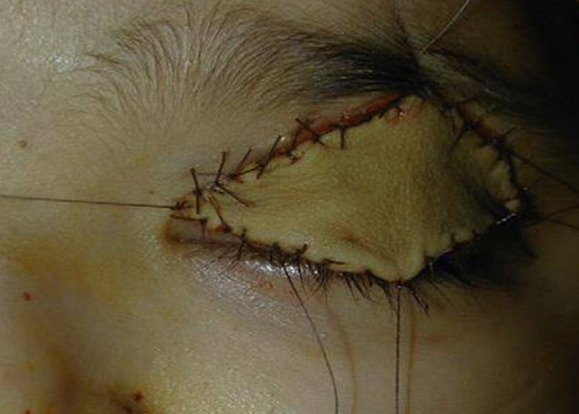
Cutaneous transplant repairing necrosis of top-left eyelid

**Figure 3:**
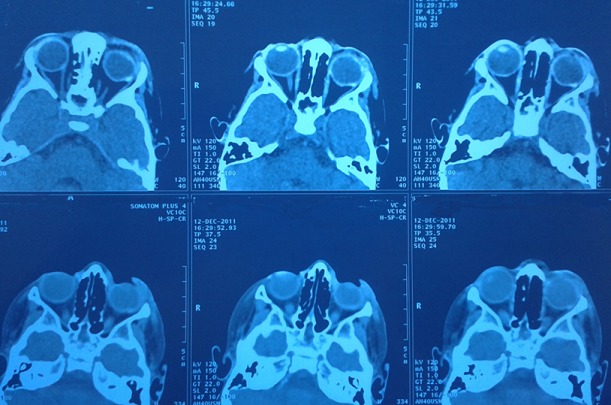
Cerebro-orbital scanner shows the left eye deformation

**Figure 4:**
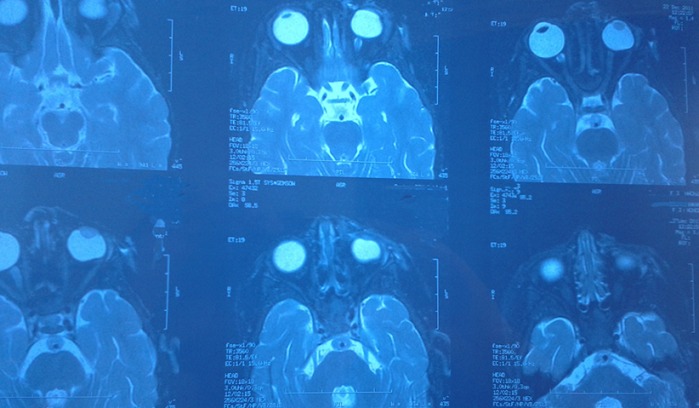
Cerebro-orbital MRI (Magnetic Resonance Imaging) shows the retraction of the left eyeball associated to ipsilateral cataract

The toxicological study of the product used for local anesthesia was performed confirming the presence of formalin in the vials and in the cupule. The Formalin was injected in the upper eyelid instead of the Lidocain (local anesthetic) and a careless medical fault was retained. The postoperative course was marked by the occurrence of necrosis of the left upper eyelid (Figure 1), deformation of the peri-orbital region associated with a retraction of the eyeball and a homolateral cataract. Despite many surgical repairs (Figure 2), the infant keeps a permanent blindness, an esthetic damage and heavily psychotic disorders requiring a child psychiatrist support (Figure 3, Figure 4). Thus, the parents complain to the penal and the civil court alleging medical malpractice and seek a legal remedy from the court to repair economic and non economic damage.

## Discussion

### Medical discussion

Formalin is a complex substance composed mainly of Formaldehyde (almost 40%) which is an organic compound and the simplest form of aldehyde. In view of its widespread use, exposure to formaldehyde is significant for human health because of its acute and chronic toxicity. To the Formaldehyde we add the methanol (8 to12%) which is a substance well known for its toxic properties. This association explains the systemic toxicity of this substance, with a metabolic acidosis which depends on the exposure's quantity. Fortunately, in our case the injected quantity was too little to pass to the systemic circulation. However, the injection of formalin in the upper eyelid had heavily loco-regional consequences in the vision prognosis with a skin necrosis, eyeball retraction and a cataract which occasioned a definite blindness. This damage is explained by the extreme reactivity of formalin with many cellular constituents and physiologic metabolites mainly with the formaldehyde (with Amino Acids, Peptides, Proteins, Nucleic Acid Bases, Nucleosides, Protein-Free DNA, Isolated nucleo-histone and with cell nuclei) [1, 8].

Once injected, the formalin fixed quickly nucleic acid and amino groups of proteins causing congestive lesions which progress to hemorrhagic lesions and finally to a local cells' lysis. At this stage, inflammatory phenomena and hypersensitivity are important. Immunologic or allergic responses in humans can be induced which can cause anaphylactic shock [5]. Loco-regional extensive necrosis of tissue is inevitable and spreads in oil spill. It is associated with infection which can cause sepsis and septic shock. After systemic absorption, Formalin is metabolized by the liver into ferric acid which is then excreted by the kidneys. Many enzymes catalyze this reaction mainly the formaldehyde dehydrogenase (FDH). (Cross links with cysteine and the sulfhydryl are formed).

Thus, the limitation of the toxicity of formaldehyde could be achieved by the administration of N-Acetyl-Cysteine [9–10]. The injection of formalin can cause three types of lesions; local caustic lesions (allergic reaction, lyses and tissue necrosis and local infection), multi-visceral impact and metabolic damage as a consequence of systemic passage and lesions related to the loco-regional extension of damage. These injuries depend on the amount of injected product, the quality and the rapidity of care and certain patient characteristics (age, immune status, defects, allergic background, etc …) [6, 11]. The effects on the eyeball are mainly related to the deformations of the eyeball which are due to caustic properties of formalin causing sclerosis of the connective-fibrous tissue of the sclera (which is the opaque, fibrous, protective, outer layer of the eye containing collagen and elastic fiber) and the damaging of Bruch´s membrane and mainly its fifth layer which is the the basement membrane of the chorio-capillaris. Added to that, Formalin has destructive impact on the cornea (loss of transparency and structural alteration of the connective tissue of the anterior epithelium, the Bowman´s membrane, the Struma, the posterior epithelium and Descemet´s membrane) and on the aqueous humor (composition and transparency). The cataract of the patient's eye can be explained by the fact that the formaldehyde binds quickly to the protein of the crystalline (water-soluble proteins that compose over 90% of the protein within the lens) because of its well-known affinity to amino acids [8–11]. Formalin poisoning requires early and efficient treatment: monitoring of caustic lesions with rigorous multi-local daily care to avoid infections and possible extensions of lesions to stop extension of damage. The role of surgery is either urgent during the existence of extensive lytic lesions (the injected quantity injected> 5 ml or/and the concentration of the product > 10%) or distant after stabilization of squelae, and its aim is corrective or/and restorative.

In our case, the infant lost the vision function definitely and the aim of the surgery was only restorative by a skin grafting in the peri-orbital area and by implanting a prosthetic eye (ocular prosthesis) to improve the appearance. In case of sign of systemic passage characterized by an early multi-organ failure or possible metabolic disorders, early treatment with N-Acetyl-Cysteine, which is a safety product, can be started [7, 10].

### Medico-legal discussion

The focus of medical liability in Tunisia is under the law of tort, specifically negligence or inattention's fault. When a penal pursuit is recommended by the victim, the author of the fault is sued and is exposed to a penal punishment and it is an offense under Article 218 and 219 of the Penal Code of the Tunisian republic (PCT) [12]. Moreover, the culprit of the breach is sued in the civil court for a material compensation which will be fixed by the judge based on different parameters among others the expert report which will fix a rate of permanent partial disability.

## Conclusion

This study probably analyzes for the first time the responsible handling procedures of formalin injection which was administered instead of local anesthetic as an accidental medical mistake. These local anesthetic bottles are commonly used to store many other products as alcohol, dental acrylic monomer, formalin, and so forth. This poses a great risk of mistaking the chemical for the local anesthesia solution. In this case, the practitioner had stored the formalin in an emptied local anesthesia bottle for the aseptic of the surgical material and to preserve eventual biopsy. His assistant loaded the formalin solution that was stored in the local anesthesia bottle because of lack of knowledge and awareness of the drug. Chemical products have the same color in the syringe, the practitioner didn't pay attention and injected the content and this is the reason of this awful event. Unfortunately, this intentional mistake has very heavy consequences because of the age of the patient, the sensitive area, the extreme reactivity of formalin with many cellular constituents and physiologic metabolites. This article emphasizes the possible risk of wrapping of certain medical consumables in a local anesthesia Therefore, with this observation we underline some malpractice and condition of storage and packaging which increase the chances of mixing up of stored toxic fluids. In order to prevent such mishaps, practitioners must pay more attention to details relative to the stock management of drugs and medical supply, otherwise, the impact would be disastrous implication not only on patients' health, but also serious penal and legal implications.
